# Biochemical characterization of New Delhi metallo-β-lactamase variants reveals differences in protein stability

**DOI:** 10.1093/jac/dku403

**Published:** 2014-10-16

**Authors:** Anne Makena, Jürgen Brem, Inga Pfeffer, Rebecca E. J. Geffen, Sarah E. Wilkins, Hanna Tarhonskaya, Emily Flashman, Lynette M. Phee, David W. Wareham, Christopher J. Schofield

**Affiliations:** 1 Department of Chemistry, University of Oxford, 12 Mansfield Road, Oxford OX1 3TA, UK; 2 Antimicrobial Research Group, Queen Mary University London, London E1 2AT, UK

**Keywords:** β-lactams, antibiotic resistance, cephalosporins, carbapenemases, thermal stability

## Abstract

**Objectives:**

Metallo-β-lactamase (MBL)-based resistance is a threat to the use of most β-lactam antibiotics. Multiple variants of the New Delhi MBL (NDM) have recently been reported. Previous reports indicate that the substitutions affect NDM activity despite being located outside the active site. This study compares the biochemical properties of seven clinically reported NDM variants.

**Methods:**

NDM variants were generated by site-directed mutagenesis; recombinant proteins were purified to near homogeneity. Thermal stability and secondary structures of the variants were investigated using differential scanning fluorimetry and circular dichroism; kinetic parameters and MIC values were investigated for representative carbapenem, cephalosporin and penicillin substrates.

**Results:**

The substitutions did not affect the overall folds of the NDM variants, within limits of detection; however, differences in thermal stabilities were observed. NDM-8 was the most stable variant with a melting temperature of 72°C compared with 60°C for NDM-1. In contrast to some previous studies, *k*_cat_/*K*_M_ values were similar for carbapenem and penicillin substrates for NDM variants, but differences in kinetics were observed for cephalosporin substrates. Apparent substrate inhibition was observed with nitrocefin for variants containing the M154L substitution. In all cases, cefoxitin and ceftazidime were poorly hydrolysed with *k*_cat_/*K*_M_ values <1 s^−1^ μM^−1^.

**Conclusions:**

These results do not define major differences in the catalytic efficiencies of the studied NDM variants and carbapenem or penicillin substrates. Differences in the kinetics of cephalosporin hydrolysis were observed. The results do reveal that the clinically observed substitutions can make substantial differences in thermodynamic stability, suggesting that this may be a factor in MBL evolution.

## Introduction

Infectious diseases remain a major public health problem worldwide. However, the utility of antimicrobial chemotherapy is compromised by the spread of resistant strains.^1^ Since their introduction over seven decades ago, the β-lactam antibiotics have been preferred antibiotics due to their high efficacy, affordability and low toxicity. Presently, β-lactams constitute >60% of antibiotics marketed worldwide.^2,3^ β-Lactamases are the most important type of resistance to β-lactam antibiotics and catalyse the hydrolysis of the β-lactam ring, rendering the antibiotics inactive.^4,5^ β-Lactamases can be classified into those that utilize an active site serine residue [serine-β-lactamases (SBLs)] or zinc ions [metallo-β-lactamases (MBLs)] in promoting the hydrolytic step in catalysis.^6,7^ From a clinical perspective, MBLs pose an increasing public health risk; they catalyse the hydrolysis of virtually all known β-lactam antibiotics except monobactams and are not inactivated by SBL inhibitors, resulting in a limited range of treatment options.^8–11^

**Figure 1.**
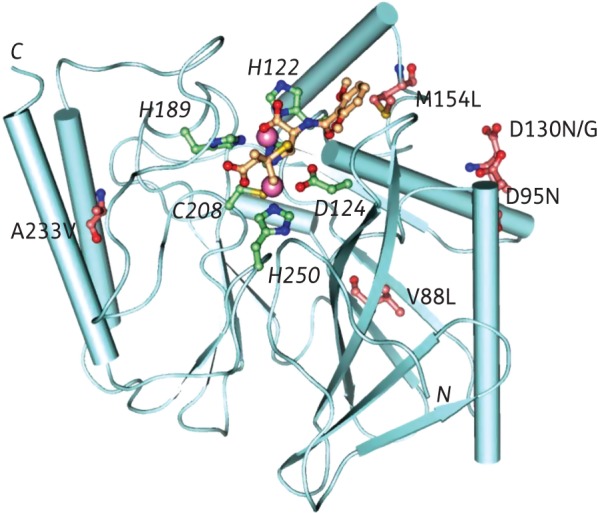
View from an NDM-1 crystal structure with hydrolysed methicillin (PDB code: 4EY2) showing the positions of the identified substitutions relative to the active site: NDM-3, D95N; NDM-4, M154L; NDM-5, V88L and M154L; NDM-6, A233V; NDM-7, D130N and M154L; and NDM-8, D130G and M154L. Pink spheres indicate the two zinc ions.

The New Delhi MBL (NDM)-1 is a clinically significant MBL encoded by the *bla*_NDM-1_ gene. NDM-1 was initially identified in 2008 in a *Klebsiella pneumonia* isolate.^12^ Since then, *bla*_NDM-1_ genes have been identified in various pathogenic bacteria, including *Escherichia coli*, *Acinetobacter baumannii* and *Pseudomonas aeruginosa*.^13–15^ The rapid global dissemination of NDM-1 and its spread to unrelated bacterial isolates via mobile genetic elements has the potential to substantially undermine β-lactam-based antibacterial chemotherapy. At the onset of this work, eight NDM variants had been described, differing from each other by one or two residues (Figure S1, available as Supplementary data at *JAC* Online). NDM-2, which has been widely reported in the Middle East, has the P28A substitution, which occurs in the predicted N-terminal periplasmic signal peptide.^16,17^ NDM-3 (D95N), NDM-4 (M154L) and NDM-6 (A233V) have a single substitution present relatively far from the zinc binding site;^18–21^ NDM-5, -7 and -8 are double-mutants containing the M154L substitution as well as the V88L, D130N and D130G substitutions, respectively (Figure 1 and Table S1).^22–24^ Genetic characterization of the NDM-1 mutants has yielded information on other resistance genes that co-harbour with *bla*_NDM_ as well as mobile genetic elements responsible for the spread of resistance.^25–27^ The biochemical characterization of NDM variants is interesting as it may provide insights into the catalytic properties of variants, the inhibition of which is desirable in the development of MBL inhibitors with a sufficient breadth of selectivity for clinical use.^19,24^

Substitutions in MBLs can cause changes in stability and/or activity; in some cases the latter are proposed to confer selective advantage during evolution of drug resistance.^28,29^ Previous biochemical analyses on NDM-3, -4 and -8 have reported different results, e.g. NDM-4^19^ is reported to have increased hydrolytic activity towards carbapenems and some cephalosporins whilst NDM-3^21^ and -8^24^ are reported to have similar or lower *k*_cat_/*K*_M_ values for various β-lactams. To address the question of whether the NDM variants have different biochemical properties, we carried out comparative studies on the substrate selectivity and thermal stabilities of seven NDM variants.

## Materials and methods

### Cloning and MIC analysis of NDM variants in *E. coli*

Genes encoding the NDM variants NDM-1, -2, -4, -5, -6 and -7 were amplified using DNA templates prepared from NDM-producing clinical isolates or *E. coli* transconjugants.^22–24^ Both full coding sequences and the NDM gene with the native IS*Ab125* promoter^22^ were amplified and cloned in the pCR-Blunt II TOPO vector (Invitrogen, Paisley, UK) and transformed in *E. coli* TOP10. The susceptibility of the transformants containing pCR2.1 NDM and pCR2.1 NDM P+ plasmids to ampicillin, cefalotin, cefoxitin, ceftazidime, ertapenem, imipenem, meropenem and doripenem was determined using the Etest method (bioMérieux, Basingstoke, UK) on Mueller–Hinton agar.

### Mutagenesis

The reported pTriEx-based pOPIN-F NDM-1 plasmid, encoding the ΔN42 NDM-1 construct (amino acids G42-R270) and a cleavable N-terminal His_6_-tag, was used as a template for site-directed mutagenesis.^30^ The truncated NDM-1 construct, lacking the NDM-1 periplasmic targeting sequence, was used due to its relative stability and activity.^31^ Primers for site-directed mutagenesis (Table S2) were from Sigma-Aldrich (Poole, UK). Site-directed mutagenesis PCR was carried out employing the Stratagene QuikChange^®^ method. A plasmid encoding for the NDM-4 variant (with the M154L substitution) was generated, and then used as a template for the production of the NDM-5, -7 and -8 variants.

### Protein production

The resultant plasmids were transformed into *E. coli* BL21 (DE3) pLysS cells for protein production; cells were cultured in modified auto-induction media.^32,33^ Protein purification was carried out by affinity chromatography and gel filtration as reported.^34^ The N-terminal His_6_-tag was cleaved using recombinant human Rhinovirus 3C Protease, and the untagged protein further purified by affinity chromatography. The purity of the resulting proteins was ascertained by SDS–PAGE (Figure S2); purified proteins were concentrated by centrifugal ultrafiltration to give a protein concentration of 15–25 mg/mL as determined by absorbance measurements at 280 nm using calculated extinction coefficients.

### MS

For LC-MS a Waters Micromass LCT Premier™ time-of-flight mass spectrometer and electrospray ionization were used. Waters MassLynx™ version 4.1 was used for data analysis (see Section 3 of the Supplementary data). The resulting combined positive ion series was deconvoluted using a maximum entropy algorithm (Figure S3). The observed masses were verified by comparison with the predicted masses obtained using the ExPasy ProtParam tool (Table S3).

### Steady-state kinetics

The hydrolysis of various β-lactam substrates was monitored at 25°C in 50 mM HEPES buffer (pH 7.2) supplemented with 1 μg/mL BSA, 1 μM ZnSO_4_ and 0.01% Triton X-100.^35^ For ampicillin hydrolysis, 50 mM MOPS buffer (pH 7.2) was used due to the high background hydrolysis of this penicillin in HEPES buffer.^36^ Analyses were carried out in triplicate (*n *≥ 3); the absorbance values were read using a BMG Labtech Pherastar FS plate reader. Extinction coefficients were determined by plotting the absorbance units against increasing concentrations of the substrates or product (Table S4). Kinetic constants (*K*_M_ and *k*_cat_) were obtained by determining the initial rate of the reaction at different substrate concentrations. The concentration-dependence of the initial rate was fitted and analysed using GraphPad Prism^®^ 5.01 software to generate Michaelis–Menten and substrate inhibition curves (Figure S4).

### Differential scanning fluorimetry (DSF)

For DSF assays a MiniOpticon™ Real-Time PCR Detection System (Bio-Rad) was used. SYPRO^®^ Orange Protein Gel Stain (Life Technologies Corporation) was used to analyse non-specific binding to hydrophobic residues; the increase in fluorescence was monitored as a function of temperature.^37^ Fluorescence readings (492 nm excitation and 610 nm emission) were taken in triplicate between 25°C and 80°C, increasing the temperature linearly in steps of 1°C/min (see Section 5 of the Supplementary data for details). Melting curves for each triplicate dataset were exported into GraphPad Prism^®^ 5.01 software, and a Boltzmann curve was fitted to determine melting temperature values (Figure S5).

### Circular dichroism (CD)

CD measurements were carried out using a Chirascan CD spectrometer (Applied Photophysics model) equipped with a Peltier temperature-controlled cell holder. Experiments were performed at 23°C in a 0.1 cm pathlength cuvette using 0.2 mg/mL protein in 10 mM sodium phosphate buffer (pH 8.0) supplemented with 50 μM ZnSO_4_. Data were recorded from 260 to 185 nm at 0.5 nm intervals; each data point was averaged for 1 s. Spectra were baseline corrected and smoothed using the Savitzky–Golay filter. Data recorded in the 190–240 nm range were analysed using DichroWeb;^38^ the CDSSTR deconvolution method was used to estimate secondary structural content using reference set 4.^39^ To minimize the effects of differences in protein concentration, the data were normalized at 207 nm.^40^

Thermal denaturation profiles were monitored by CD at 222 nm, with data recorded every 1°C from 10 to 90°C at a ramp-rate of 1°C/min. Normalized data were fitted to a Boltzmann sigmoidal curve in GraphPad Prism^®^ 5.01 software to determine melting temperature values. Spearman's rank correlation coefficient was used to compare the data from DSF with the temperature-dependent CD results to determine their correlation (Table S5). The correlation analysis was carried out using StatsDirect (http://www.statsdirect.com/).

## Results

### Comparative analysis of the β-lactam susceptibility of the NDM variants in *E. coli*

Due to the variations in the reported MIC values for NDM variants from different studies^19,21–24^ (Table S6), we tested the antibiotic susceptibility of the reported NDM variants using the same expression system. Differences in the effects of the NDM variants on the susceptibility of *E. coli* to a range of β-lactams were assessed in *E. coli* TOP10 cells that were transformed with plasmids containing NDM genes cloned with and without the native promoter. Consistent with previous reports,^22,23^ expression from the native (IS*Aba125*) promoter resulted in >4-fold higher MICs of ertapenem, imipenem and doripenem in *E. coli* TOP10 (Table 1). Almost all of the transformants were resistant to ampicillin, cefalotin, cefoxitin and ceftazidime (MIC >256 mg/L) with both native and T7 promoters. However, differences in the susceptibility of the variants to carbapenems were clearly observed when the genes were expressed under the native promoter (Table 1 and Figure S6). Constructs containing NDM-4, -5 and -7 displayed >4-fold higher MIC values of imipenem compared with NDM-1, -2 and -6 (for expression from the native promoter), and the values were also higher than those reported for NDM-3 and -8 (transformed in *E. coli* DH5α).^21,24^ We therefore investigated whether the observed differences reflect changes in the biochemical properties of the variants by studies on the recombinant enzymes.

**Table 1.** Susceptibility of *E. coli* transformed with plasmids containing NDM variants

Host strain	Plasmid^a^	MIC determined by Etest (mg/L)							
		AMP	CEF	FOX	CAZ	ERT	IMP	DOR	MEM
*E. coli* TOP10	pCR2.1	4	0.008	0.008	0.125	0.008	0.25	0.032	0.047
*E. coli* TOP10	pCR2.1 NDM-1	>256	>256	24	>256	0.38	0.38	0.25	0.38
*E. coli* TOP10	pCR2.1 NDM-1 P+	>256	>256	>256	>256	8	8	8	4
*E. coli* TOP10	pCR2.1 NDM-2	>256	>256	>256	>256	2	0.38	0.38	0.38
*E. coli* TOP10	pCR2.1 NDM-2 P+	>256	>256	>256	>256	16	8	8	4
*E. coli* DH5α	pHSG398/NDM-3^b^	256	NA	32	256	NA	0.25	0.125	0.25
*E. coli* TOP10	pCR2.1 NDM-4	>256	>256	>256	>256	2	0.25	0.032	0.38
*E. coli* TOP10	pCR2.1 NDM-4 P+	>256	>256	>256	>256	16	>32	12	8
*E. coli* TOP10	pCR2.1 NDM-5	>256	>256	>256	>256	2	0.25	0.5	0.38
*E. coli* TOP10	pCR2.1 NDM-5 P+	>256	>256	>256	>256	>32	>32	12	>32
*E. coli* TOP10	pCR2.1 NDM-6	>256	>256	>256	>256	1	0.38	0.125	0.38
*E. coli* TOP10	pCR2.1 NDM-6 P+	>256	>256	>256	>256	>32	8	1.5	>32
*E. coli* TOP10	pCR2.1 NDM-7	>256	>256	>256	32	2	2	0.5	1
*E. coli* TOP10	pCR2.1 NDM-7 P+	>256	>256	>256	>256	>32	>32	8	>32
*E. coli* DH5α	pHSG398/NDM-8^b^	256	NA	64	256	NA	0.5	NA	0.25

AMP, ampicillin; CEF, cefalotin; FOX, cefoxitin; CAZ, ceftazidime; ERT, ertapenem; IMP, imipenem; DOR, doripenem; MEM, meropenem; NA, not available.

^a^P+ indicates plasmids containing the native IS*Aba125* promoter.

^b^Data from Tada *et al*.^21,24^ MICs determined by broth microtitre dilution.

### NDM variants present similar structural properties

**Figure 2.**
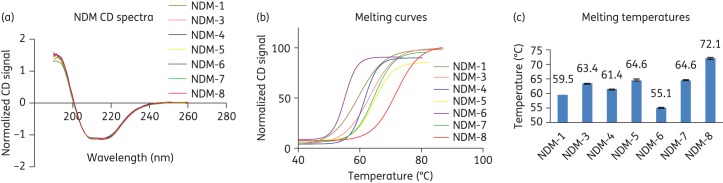
Clinically observed substitutions alter the stability of NDM variants. (a) Normalized CD spectra of NDM variants. (b) Thermal stability melting curves. (c) Melting temperatures of the NDM variants as determined by CD: NDM-1, 59.5 ± 0.1; NDM-3, 63.4 ± 0.1; NDM-4, 61.4 ± 0.2; NDM-5, 64.6 ± 0.3; NDM-6, 55.1 ± 0.3; NDM-7, 64.6 ± 0.2; and NDM-8, 72.1 ± 0.4. Data are the means of triplicate experiments, with error bars showing the standard deviation (±SD).

To investigate the biochemical effects of clinically reported NDM variants, we generated seven NDM variants by site-directed mutagenesis. A three-step chromatography-based purification procedure yielded the active NDM variants with >90% purity (by SDS–PAGE). All the variants were expressed at similar levels in the growth conditions used. Mass spectrometric analyses by LC-MS verified the masses of the recombinant proteins, which were all in close agreement with the calculated values (Table S3). The secondary structure of the variants was then investigated using CD spectroscopy at 23°C. The CD spectra for all tested variants were characteristic of well-folded, structured proteins;^41^ the β-sheet and α-helical content from the deconvolution were in agreement with the crystallographically observed structural features of NDM-1.^17,30^ Despite the observation of slight differences in the 190 nm region, the CD spectra and predicted secondary structure content of the wild-type and the tested NDM variants were similar, suggesting that the substitutions do not substantially affect the overall folds of the NDM variants (Figure 2a).

### Investigation of kinetic parameters of NDM variants

We then investigated the roles of the clinically observed substitutions in NDM catalysis. Steady-state kinetic parameters were determined for the variants against a representative set of carbapenem, penicillin and cephalosporin β-lactam antibiotic substrates.

The enzymes hydrolysed all the tested carbapenems, as do other MBLs (Table 2). There was no evidence for substantial differences in catalytic efficiencies of the variants with carbapenems as reflected in *k*_cat_/*K*_M_ values, with the largest differences observed being in the 5-fold range, i.e. NDM-6 has ∼5-fold higher *k*_cat_/*K*_M_ values compared with NDM-1 and -8, respectively, for doripenem. Somewhat larger differences in the separate *k*_cat_ and *K*_M_ values were observed (e.g. NDM-8 has an 11-fold decrease in *k*_cat_ compared with NDM-1 for imipenem). Our results are therefore not consistent with a recent study^21^ reporting consistently lower *k*_cat_/*K*_M_ values for NDM-3 compared with NDM-1 with a similar set of substrates.

**Table 2.** Kinetic parameters of seven NDM variants with representative β-lactam antibiotics

Antibiotic	NDM-1	NDM-3	NDM-4	NDM-5	NDM-6	NDM-7	NDM-8
Carbapenems							
imipenem							
*K*_M_ (µM)	78 ± 4	82 ± 12	62 ± 8	148 ± 16	62 ± 6	56 ± 6	20 ± 3
*k*_cat_ (s^−1^)	600	757	252	332	355	127	54
*k*_cat_/*K*_M_ (s^−1^ µM^−1^)	7.6	9.2	4.1	2.2	5.8	2.3	2.9
meropenem							
*K*_M_ (μM)	57 ± 9	58 ± 11	119 ± 9	99 ± 4	49 ± 4	45 ± 5	54 ± 5
*k*_cat_ (s^−1^)	301	238	583	413	362	194	141.7
*k*_cat_/*K*_M_ (s^−1^ μM^−1^)	5.2	4.2	4.9	4.1	7.4	4.3	2.6
doripenem							
*K*_M_ (μM)	119 ± 17	83 ± 15	125 ± 10	126 ± 11	119 ± 18	64 ± 11	151 ± 9
*k*_cat_ (s^−1^)	226	170	743	570	1032	348	267.4
*k*_cat_/*K*_M_ (s^−1^ μM^−1^)	1.9	2.0	6.0	4.5	8.6	5.4	1.7
Penicillins							
ampicillin							
*K*_M_ (μM)	110 ± 23	258 ± 54	305 ± 48	156 ± 20	455 ± 69	207 ± 55	229 ± 45
*k*_cat_ (s^−1^)	447	724	900	408	1375	477	273
*k*_cat_/*K*_M_ (s^−1^ μM^−1^)	4.1	2.8	2.9	2.6	3.0	2.3	1.2
Cephalosporins							
cefalotin							
*K*_M_ (μM)	29 ± 6	13 ± 3	11 ± 2	15 ± 3	10 ± 2	11 ± 3	5 ± 1
*k*_cat_ (s^−1^)	75	53	45	25	63	18	9
*k*_cat_/*K*_M_ (s^−1^ μM^−1^)	2.6	4.6	4.1	1.6	6.1	1.5	1.9
cefoxitin							
*K*_M_ (μM)	43 ± 6	50 ± 6	63 ± 7	27 ± 5	107 ± 9	23 ± 3	16 ± 3
*k*_cat_ (s^−1^)	10	6	14	4	18	4	3
*k*_cat_/*K*_M_ (s^−1^ μM^−1^)	0.23	0.13	0.22	0.14	0.17	0.15	0.18
ceftazidime							
*K*_M_ (μM)	100 ± 19	115 ± 15	133 ± 18	86 ± 11	144 ± 25	79 ± 10	19 ± 5
*k*_cat_ (s^−1^)	24	23	28	15	29	16	5
*k*_cat_/*K*_M_ (s^−1^ μM^−1^)	0.23	0.20	0.21	0.18	0.20	0.20	0.26
nitrocefin							
*K*_M_ (μM)	11 ± 2	4 ± 1	4 ± 1	6 ± 1	8 ± 2	5 ± 1	4 ± 1
*K*_i_ (μM)	NR	NR	102 ± 32	139 ± 28	NR	79 ± 18	146 ± 47
*k*_cat_ (s^−1^)	65	30	38	33	49	15	44
*k*_cat_/*K*_M_ (s^−1^ μM^−1^)	5.9	7.5	6.3	5.6	6.1	3.1	10.9

NR, not reported.

Measurements were carried out in triplicate (*n*≥3) using a single batch of enzyme; *K*_M_ values are the means of three independent measurements ± standard deviation. Standard deviation values for *k*_cat_ did not exceed 10%.

The tested penicillin substrate (ampicillin) was readily hydrolysed by all of the NDM variants. However, for all of the variants the *k*_cat_/*K*_M_ values were lower than for NDM-1. Except for NDM-8 (the least active variant with ampicillin), the apparently elevated *K*_M_ values are, in part, compensated for by increased *k*_cat_ values.

There were evident differences in the kinetic parameters for the tested cephalosporins. Although the differences in *k*_cat_/*K*_M_ values for the cephalosporins were at most 3–4-fold, there were substantial differences in the separate *k*_cat_ and *K*_M_ values, e.g. NDM-8 shows an 8-fold lower *k*_cat_ for cefalotin than NDM-1. Indeed, the doubly substituted variants NDM-5 (V88L, M154L), NDM-7 (D130N, M154L) and NDM-8 (D130G, M154L) showed consistently lower *k*_cat_ values for the tested cephalosporins except for nitrocefin. There were also differences in the *K*_M_ values of the NDM variants with the tested cephalosporins, with the variants having low *K*_M_ values for nitrocefin and cefalotin. Unlike other variants, NDM-8 showed a distinctly lower *K*_M_ for ceftazidime (∼7-fold lower than NDM-4 and -6). Cefoxitin and ceftazidime were relatively poorly hydrolysed by NDM-1 and all variants, with *k*_cat_/*K*_M_ values being <1 s^−1^ μM^−1^ for all of the variants, consistent with work on ceftazidime and cefotaxime resistance by NDM-1-producing *E. coli* cells.^42^

Notably, apparent substrate inhibition was observed for nitrocefin with NDM-4, -5, -7 and -8 (*K*_i_ values of 102 ± 32, 139 ± 28, 79 ± 18 and 146 ± 47 μM, respectively). Nitrocefin substrate inhibition was not observed for NDM-1 or the other tested NDM variants, or the other tested cephalosporins.

### NDM variants display differences in thermal stability

Although, we did not observe substantial differences in the kinetic parameters for carbapenem hydrolysis by the NDM variants, the positions of some of the substitutions in the MBL fold (Figure 1) suggested that they may affect the biophysical properties of the variants. We therefore investigated the effects of the substitutions on the stability of the variants using temperature-dependent CD. In contrast to (most of) the kinetic analyses, the CD results revealed clear differences in the thermal stabilities of the NDM variants (Figure 2b). In general, the NDM variants containing two substitutions were found to be more stable to thermal denaturation compared with the single-substituted variants (Figure 2c), suggesting that ‘second substitutions’ may be involved in stabilization. The doubly substituted NDM-8 (D130G, M154L) had the highest melting temperature at 72°C while both NDM-5 (V88L, M154L) and NDM-7 (D130N, M154L) had a melting temperature of about 65°C compared with NDM-1, which had a melting temperature of 60°C (Figure 2c). Singly substituted NDM-6 (A233V) was the least stable variant with a melting temperature of 55°C, while NDM-3 (D95N) and NDM-4 (M154L) had melting temperature values of 63 and 61°C, respectively. These results were corroborated by DSF melting temperature-shift analysis. According to the DSF analyses (Figure S5), the NDM variants exhibited higher melting temperatures in HEPES buffer. To investigate the stabilizing effect of Zn (II) ions, a comparison was carried out in the absence or presence of 50 μM ZnCl_2_. In both buffers, addition of zinc ions stabilized the NDM variants with the exception of NDM-5. The relative stabilizing effect of HEPES buffer was less apparent in the presence of zinc ions, as similar melting temperature values were recorded in both buffers in the presence of 50 μM ZnCl_2_. Spearman's rank correlation coefficient analysis (ρ = 0.76, *P* < 0.05), indicates a strong positive correlation between the DSF and CD data (Table S5).

## Discussion

Selective pressure caused by increased use of carbapenems, specifically imipenem, has been suggested to drive evolution of MBLs, including variants of the IMP and VIM MBLs.^43^ Considering both our work and that of others,^19,21,24^ as well as the differences arising from the use of different procedures, the small differences (3–5-fold) observed in reported *k*_cat_/*K*_M_ values for NDM variants with carbapenem substrates may be within error. The variations observed between our work and literature values and between different literature studies^19,21,24^ (Table S7) could reflect differences in enzyme preparation procedures and assay conditions, which may influence purity, protein folding and metal content. In contrast to the proposals of others,^19^ our current view is that, whilst the observed substitutions likely do have effects on the kinetic parameters with some substrates, there is as yet no compelling evidence that the studied variants have evolved to directly increase kinetic parameters for carbapenem hydrolysis.

Despite this conclusion, our results do reveal kinetic differences between the NDM variants, notably in that some, but not all, display substrate inhibition kinetics with nitrocefin. Nitrocefin substrate inhibition has been reported for IMP MBL variants with substitutions relatively remote from the active site (S121G and F218Y).^44^ In the case of the NDM variants, nitrocefin substrate inhibition was only observed in variants with the M154L substitution (NDM-4, -5, -7 and -8), suggesting that it results from a specific interaction. Although nitrocefin is not used clinically, these results do reveal the potential for clinically observed NDM variants to have different kinetic properties with different β-lactams. One possibility is that the M154L substitution alters interactions between residue Met-154 and the nitrocefin dinitroaryl-substituent slowing catalysis (Figure S7). These observations may be useful in work on the development of MBL inhibitors and β-lactam antibiotics with reduced susceptibility to MBL catalysis.

The substitutions present in NDM variants did not alter the overall structural composition of the enzymes as indicated by their CD spectra. However, the variants showed differences in their stabilities with respect to thermal denaturation as determined by CD and DSF analyses, with >10°C differences in melting temperature values being observed in some cases. Notably, the variants with higher melting temperature values, i.e. doubly substituted NDM-5, -7 and -8, were less catalytically active in comparison with the variants with lower melting temperature values such as NDM-6. The detailed structural reasons for the observed differences in stability and their potential relationship to differences in catalytic properties are as yet not apparent. However, from a practical perspective, it is important that the potential differences in thermodynamic stability of the NDM variants, and possibly other MBLs, are taken into account in future kinetic studies of NDM variants, including in inhibition studies.

According to the antibiotic susceptibility profiles of the NDM variants, the more stable variants, NDM-5 and -7, did show an increase in MIC values of selected carbapenems in comparison with the less stable variants. However, the biological relevance, if any, of the different stabilities of NDM variants is as yet unclear. It should be noted that the NDM enzymes are mostly found in Enterobacteriaceae that normally live (at least in humans) at ∼37°C, which is below the melting temperature values for all the studied variants (≥55°C). Other than non-functionally related evolutionary drift (which cannot be entirely excluded), it is possible that the differences in thermodynamic stabilities reflect environmental pressures (including temperature variations) on bacteria harbouring specific NDM variants. In the case of the SBLs, it is proposed that, at least in some cases, the evolution of improved catalytic efficiency with ‘new’ substrates can come at a cost with respect to decreased thermodynamic stability.^45,46^ However, as yet, there is no evidence for such a relationship with the known NDM variants. It is also possible that the *relative* differences in stability reflect longer lifetimes in cells, resulting in elevated MIC values. This could be due to increased thermodynamic stability, a decreased propensity to aggregate under *in vivo* conditions and/or increased stability with respect to protease-mediated degradation, which can, but does not necessarily, correlate with thermodynamic stability.^47^

## Funding

This research was supported by: the Rhodes Trust (UK); a Clarendon Scholarship; a St Hugh's College W. Louey Scholarship; the Biotechnology & Biological Sciences Research Council (BBSRC); a Royal Society Dorothy Hodgkin Research Fellowship; and the Medical Research Council (MRC)/Canadian Grant G1100135.

## Transparency declarations

None to declare.

## Supplementary data

Supplementary data, including Figures S1–S7 and Tables S1–S7, are available at *JAC* Online (http://jac.oxfordjournals.org/).

Supplementary Data
