# Supplementary material for: Biochemical characterization of New Delhi metallo-β-lactamase variants reveals differences in protein stability
Source: J Antimicrob Chemother. 2014 Oct 16;70(2):463–9. doi: 10.1093/jac/dku403 (PMC4291237; doi:10.1093/jac/dku403)
Supplement: Supplementary Data [file supp_70_2_463__index.html]

Biochemical characterization of New Delhi metallo-β-lactamase variants reveals differences in protein stability — Supplementary Data 

# Biochemical characterization of New Delhi metallo-β-lactamase variants reveals differences in protein stability

## Supplementary Data

Supplementary Data

**Files in this Data Supplement:**

- Supplementary Data - Doc file
