# Supplementary material for: Biochemical characterization of New Delhi metallo-β-lactamase variants reveals differences in protein stability
Source: J Antimicrob Chemother. 2014 Oct 16;70(2):463–9. doi: 10.1093/jac/dku403 (PMC4291237; doi:10.1093/jac/dku403)
Supplement: Supplementary Data [file supp_dku403_dku403supp.doc]

**Supplementary data**

Table of contents

[Section 1. NDM variants sequence comparison. 2](#__RefHeading___Toc400542742)

[Figure S1. Sequence alignment of NDM-1 to NDM-8. 2](#__RefHeading___Toc400542743)

[Table S1. Geographical distribution of reported NDM variants. 3](#__RefHeading___Toc400542744)

[Section 2. NDM-1 variant production and purification. 4](#__RefHeading___Toc400542745)

[Table S2. Primers used for generation of NDM variants by site-directed mutagenesis. 4](#__RefHeading___Toc400542746)

[Figure S2. Gel filtration chromatograms of NDM variants 4](#__RefHeading___Toc400542747)

[Section 3. Protein mass spectrometry. 5](#__RefHeading___Toc400542748)

[Figure S3. ESI-MS spectra of NDM variants. 6](#__RefHeading___Toc400542749)

[Table S3. Comparison of predicted and observed masses of the NDM variants 6](#__RefHeading___Toc400542750)

[Section 4. Kinetic analyses 7](#__RefHeading___Toc400542751)

[Table S4. Extinction coefficients of the antibiotics used. 7](#__RefHeading___Toc400542752)

[Figure S4. Kinetic analysis of NDM variants with different β-lactam antibiotics. 8](#__RefHeading___Toc400542753)

[Section 5. Differential Scanning Fluorimetry (DSF). 10](#__RefHeading___Toc400542754)

[Figure S5. Results of DSF analyses of NDM variants. 10](#__RefHeading___Toc400542755)

[Table S5. Spearman’s rank correlation analysis of DSF and CD results. 11](#__RefHeading___Toc400542756)

[Table S6. Comparison of MIC values of NDM variants 12](#__RefHeading___Toc400542757)

[Figure S6. Comparative MICs of *E. coli* TOP10 transformants 13](#__RefHeading___Toc400542758)

[Table S7. Comparison of kinetic parameters 14](#__RefHeading___Toc400542759)

[Figure S7. View from a structure of NDM-1 15](#__RefHeading___Toc400542760)

# Section 1. NDM variants sequence comparison.

## Figure S1. Sequence alignment of NDM-1 to NDM-8.

Alignment of NDM variants sequences obtained from [http://www.lahey.org/studies/other.asp#table1](http://www.lahey.org/studies/other.asp#table1) generated using ESPript 3.0^1^: NDM-2: P28A; NDM-3: D95N; NDM-4: M154L; NDM-5: V88L, M154L; NDM-6: A233V; NDM-7: D130N, M154L and NDM-8: D130G, M154L. Secondary structure elements are derived from an NDM-1 crystal structure (PDB code: 3Q6X); asterisks indicate zinc binding residues (H120, H122, H189; D124, C208, H250).


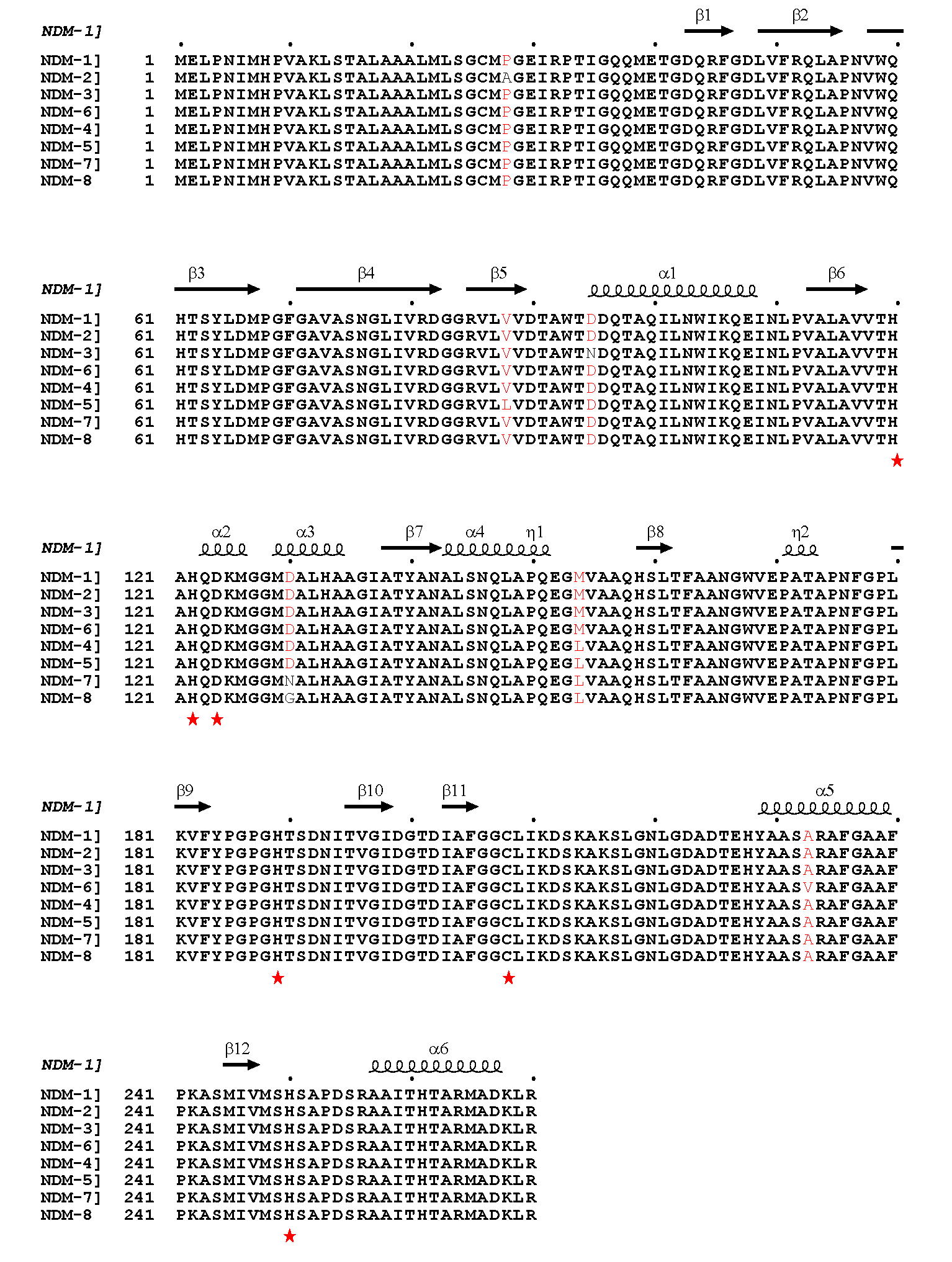


## Table S1. Geographical distribution of reported NDM variants.

| Reported NDM variants (<http://www.lahey.org/Studies/other.asp>). | | | |
| --- | --- | --- | --- |
| NDM type | Residue substitution | Bacteria species | Geographic location identified |
| NDM-2 | P28A | *A. baumannii* | Egypt, Israel and United Arab Emirates^2,3^ |
| NDM-3 | D95N | *E. coli* | Australia, Japan^4,5^ |
| NDM-4 | M154L | *E. coli, Enterobacter cloacae* | France (previous hospitalisation in Cameroun) Denmark (previously in Vietnam), Czech Republic (previous hospitalisation in Sri Lanka)^6-8^ |
| NDM-5 | V88L, M154L | *E. coli* | UK (previous hospitalisation in India)^9^ |
| NDM-6 | A233V | *E. coli* | New Zealand (previous hospitalisation in India) and USA^10,11^ |
| NDM-7 | D130N,M154L | *E. coli* | Germany (previous hospitalisation in India)  France (previous travel to Burma)^12,13^ |
| NDM-8 | D130G,M154L | *E. coli* | Nepal^14^ |
| NDM-9 | E152K | *K. pneumoniae* | China |
| NDM-10 | G69S, A74T  G200R | *K. pneumoniae* | India^a^ |
| NDM-11 | Assigned | Not available | Not available^a^ |
| NDM-12 | M154L, G222D | *E. coli* | Nepal^15^ |
| ^a^ NDM-10 and NDM-11 nucleotide sequences have been directly submitted to Genbank under accession numbers KF361506.1 and AB926431 respectively. | | | |

# Section 2. NDM-1 variant production and purification.

| Table S2. Primers used for generation of NDM variants by site-directed mutagenesis. | | | |
| --- | --- | --- | --- |
| NDM | Residue | Forward primer (5’-3’) | Reverse primer (5’-3’) |
| NDM-3 | D54N | GATACCGCATGGACCAATGATCAGACCGCAC | GTGCGGTCTGATCATTGGTCCATGCGGTATC |
| NDM-4 | M113L | ATGGTGGTCGTGTTCTGCTGGTTGATACCGCATGGAC | GCTGCAACCAGACCTTCTTGCGGAGCCAGC |
| NDM-5 | V47L | ATGGTGGTCGTGTTCTGCTGGTTGATACCGCATGGAC | GTCCATGCGGTATCAACCAGCAGAACACGACCACCAT |
| NDM-6 | V192A | ACATTATGCAGCAAGCGTACGTGCATTTGGTGCAG | CTGCACCAAATGCACGTACGCTTGCTGCATAATGT |
| NDM-7 | D89N | ACAAAATGGGTGGTATGAATGCACTGCATGCAGCA | TGCTGCATGCAGTGCATTCATACCACCCATTTTGT |
| NDM-8 | D89G | CAAAATGGGTGGTATGGGTGCACTGCATGCAGCAG | CTGCTGCATGCAGTGCACCCATACCACCCATTTTG |

Figure S2. Gel filtration chromatograms of NDM variants**.** (Elution buffer: 50mM HEPES, 200mM NaCl, pH 7.5); the insert shows SDS PAGE gel analysis after the cleavage of the *N*-terminal His_6_-tag; vertical black lines indicate NDM containing fractions.


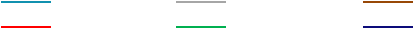


NDM-3 NDM-4 NDM-5

NDM-6 NDM-7 NDM-8

# Section 3. Protein mass spectrometry.

Mass spectrometric analyses were carried out for the ΔN42 NDM variants after cleavage of the *N*-terminal His_6_-tag. An LCT Premier mass spectrometer (Waters) was coupled to an Agilent 1100 Series HPLC using a Chromolith® FastGradient RP‑18 endcapped column equipped with a 50‑2 HPLC column, made of monolithic silica (C_18_, 2 x 50 mm, macropores with 1.6 µm diameter, *Merck*). The instrument was connected to a CTC‑autosampler inlet system. A multi‑step gradient over 10 min was run (solvent A 94.9% H_2_O/5% CH_3_CN/0.1% formic acid, solvent B 99.9% CH_3_CN/0.1% formic acid; 0‑1 min 5% B for equilibration, followed by a linear gradient to 100% B over 4 min, then 100% B for an additional 3 min, followed by a linear gradient over 2 min back to 5% B to re‑equilibrate the column) to separate the protein samples at flow rates of 0.4 ml/min for the first 5 min and then 1.0 ml/min for the remaining time. The electrospray ionisation source used a capillary voltage of 3.2 kV and cone voltage of 25 V. Nitrogen was used as the nebulizer and desolvation gas at a flow rate of 600 l/h. Protein typically eluted as a peak between 3 and 5 min under these conditions. Calculated masses were obtained using the ExPasy ProtParam tool (http://web.expasy.org/protparam/).

## Figure S3. ESI-MS spectra of NDM variants.

Combined ion series and deconvoluted mass spectra for ΔN42 NDM-1 after applying the maximum entropy algorithm to the shown ion series. Masses are in Daltons.

| Table S3. Comparison of predicted and observed masses of the NDM variants. | | |
| --- | --- | --- |
| NDM variant | Predicted mass (daltons) | Observed mass (daltons) |
| NDM-1 | 24318.4 | 24320.5 |
| NDM-3 | 24317.4 | 24319.2 |
| NDM-4 | 24300.3 | 24301.5 |
| NDM-5 | 24314.4 | 24315.5 |
| NDM-6 | 24346.4 | 24347.2 |
| NDM-7 | 24299.4 | 24300.5 |
| NDM-8 | 24242.3 | 24243.7 |

# Section 4. Kinetic analyses

| Table S4. Extinction coefficients of the antibiotics used. | | | |
| --- | --- | --- | --- |
| Calibration curves | Substrates | Ɛ AU^1^/µM | λ_Max_  (nm) |
| 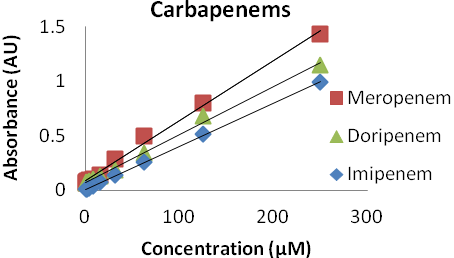 | Imipenem | 0.0040 | 300 |
|  | Meropenem | 0.0055 | 300 |
|  | Doripenem | 0.0044 | 300 |
| 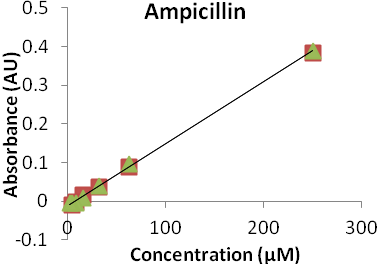 | Ampicillin | 0.0019 | 235 |
| 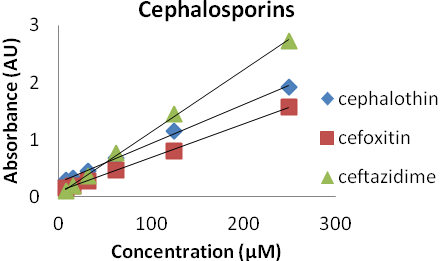 | Cephalothin | 0.0066 | 265 |
|  | Cefoxitin | 0.0070 | 265 |
|  | Ceftazidime | 0.0113 | 265 |
| 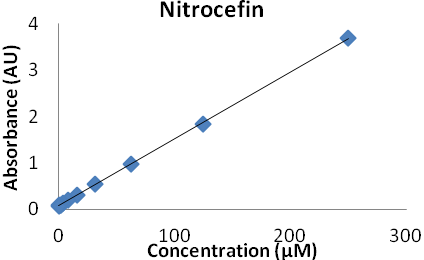 | Nitrocefin | 0.0078 | 495 |
| For calculation of the effective molar extinction coefficients, standard curves for the antibiotics were generated by reading the absorbance of the substrates at their specific wavelength using increasing concentrations of the substrate. Experiments were done in triplicate; standard deviations were <10% of the mean. ^1^AU refers to recorded absorbance units. | | | |

## Figure S4. Kinetic analysis of NDM variants with different β-lactam antibiotics.

#

#

N

S

O

OH

H

N

O

S

O

NO

2

NO

2

# Section 5. Differential Scanning Fluorimetry (DSF).

DSF assays were carried out in white 8 x 6 PCR wells. The total assay volume per well was 50 µl with a final concentration of 2 µM protein and, where appropriate, 50 µM ZnCl_2_. The SYPRO Orange Protein Gel Stain stock solution in DMSO (5000x concentrate) was diluted 1:2500 in 50 mM HEPES or Tris buffer respectively, pH 7.5, containing 200 mM NaCl. Fluorescence readings (monitored at 492 nm excitation and 610 nm emission) were taken in triplicate between 25°C and 80°C, increasing the temperature linearly in steps of 1°C every minute.

## Figure S5. Results of DSF analyses of NDM variants.

Differential Scanning Fluorimetry analysis of NDM-1 to NDM-8 represented by melting curves under the stated conditions and derived *T*_m_ values. Analyses in (a) HEPES and (b) Tris buffer without added metal, and (c) corresponding derived *T*_m_ values in each buffer without added metal. Melting curves in the presence of 50 µM ZnSO_4_ in (d) HEPES and (e) Tris buffer, and e) corresponding derived *T*_m_ values. Data are the mean of triplicate experiments, with error bars showing the standard deviation (±SD).

##

## Table S5. Spearman’s rank correlation analysis of DSF and CD results.

The analysis was carried out using the non-parametric analysis function in StatsDirect (<http://www.statsdirect.com/>).

|  | Melting temperatures (°C) | |
| --- | --- | --- |
|  | CD (Phosphate buffer + 50 µM ZnSO_4_) | DSF (**HEPES**+ 50 µM ZnCl_2_) |
| NDM-1 | 59.5 | 57.7 |
| NDM-3 | 63.4 | 60.1 |
| NDM-4 | 61.4 | 61.5 |
| NDM-5 | 64.6 | 64.5 |
| NDM-6 | 55.1 | 60.9 |
| NDM-7 | 64.6 | 65.7 |
| NDM-8 | 72.1 | 65.5 |
| Spearman’s rank correlation coefficient | | 0.75 |

|  | Melting temperatures (°C) | |
| --- | --- | --- |
|  | CD (Phosphate buffer + 50 µM ZnSO_4_) | DSF (**TRIS**+ 50 µM ZnCl_2_) |
| NDM-1 | 59.5 | 57.5 |
| NDM-3 | 63.4 | 59.6 |
| NDM-4 | 61.4 | 61.0 |
| NDM-5 | 64.6 | 64.3 |
| NDM-6 | 55.1 | 60.7 |
| NDM-7 | 64.6 | 65.4 |
| NDM-8 | 72.1 | 65.4 |
| Spearman’s rank correlation coefficient | | 0.76 |

The CD data was compared to the DSF data obtained using HEPES or Tris buffers supplemented with 50 µM ZnCl_2_. The Spearman’s rank correlation coefficient of <0.75 indicates a strong correlation between the DSF and CD datasets.

| Table S6. Comparison of MIC values of NDM variants. | | | | | | | | | |
| --- | --- | --- | --- | --- | --- | --- | --- | --- | --- |
| **Host Strain** | **Plasmid^α^** | **MIC mg/L** | | | | | | | |
|  |  | **^β^AMP** | **CEP** | **FOX** | **CAZ** | **ERT** | **IMP** | **DOR** | **MEM** |
| *E. coli* TOP10 | pCR2.1 | 4 | 0.008 | 0.008 | 0.125 | 0.008 | 0.25 | 0.032 | 0.047 |
| *E. coli* TOP10 | pCR2.1 NDM-1 | >256 | >256 | 24 | >256 | 0.38 | 0.38 | 0.25 | 0.38 |
| *E. coli* TOP10 | pCR2.1 NDM-1 P+ | >256 | >256 | >256 | >256 | 8 | 8 | 8 | 4 |
| *E. coli* TOP10^16^ | pK18 NDM-1 | >256 | >256 | >256 | >256 | 24 | 12 | ND | 12 |
| *E. coli* TOP10 | pCR2.1 NDM-2 | >256 | >256 | >256 | >256 | 2 | 0.38 | 0.38 | 0.38 |
| *E. coli* TOP10 | pCR2.1 NDM-2 P+ | >256 | >256 | >256 | >256 | 16 | 8 | 8 | 4 |
| *E. coli* DH5α^2^ | pCU19 NDM-2 | ND | ND | ND | >256 | ND | 1 | ND | 2 |
| *E. coli* DH5α^5^ | pHSG398/NDM-3 | 256 | N/A | 32 | 256 | N/A | 0.25 | 0.125 | 0.25 |
| *E. coli* TOP10 | pCR2.1 NDM-4 | >256 | >256 | >256 | >256 | 2 | 0.25 | 0.032 | 0.38 |
| *E. coli* TOP10 | pCR2.1 NDM-4 P+ | >256 | >256 | >256 | >256 | 16 | >32 | 12 | 8 |
| *E. coli* TOP10^6^ | pNDM-4 | ND | ND | 256 | 256 | 16 | 16 | 8 | 8 |
| *E. coli* TOP10 | pCR2.1 NDM-5 | >256 | >256 | >256 | >256 | 2 | 0.25 | 0.5 | 0.38 |
| *E. coli* TOP10 | pCR2.1 NDM-5 P+ | >256 | >256 | >256 | >256 | >32 | >32 | 12 | >32 |
| *E. coli* TOP10^9^ | pCR2.1 NDM-5 P+ | ND | ND | >256 | >256 | 32 | >32 | ND | 32 |
| *E. coli* TOP10 | pCR2.1 NDM-6 | >256 | >256 | >256 | >256 | 1 | 0.38 | 0.125 | 0.38 |
| *E. coli* TOP10 | pCR2.1 NDM-6 P+ | >256 | >256 | >256 | >256 | >32 | 8 | 1.5 | >32 |
| *E. coli* ARL10/167 | pNDM-6 ( from clinical isolate) | ND | ND | ND | ND | >32 | >32 | >32 | >32 |
| *E. coli* TOP10 | pCR2.1 NDM-7 | >256 | >256 | >256 | 32 | 2 | 2 | 0.5 | 1 |
| *E. coli* TOP10 | pCR2.1 P+NDM-7 | >256 | >256 | >256 | >256 | >32 | >32 | 8 | >32 |
| *E. coli* DH10B^13^ | pCR Blunt-II TOPO NDM-7 | ND | ND | >256 | >256 | 16 | 16 | ND | 8 |
| *E. coli* TOP10^12^ | pCR-Blunt II-TOPO pNDM-7 | >256 | ND | ND | >256 | >32 | >32 | 8 | >32 |
| *E. coli* DH5α^14^ | pHSG398/NDM-8 | 256 | N/A | 64 | 256 | N/A | 0.5 | N/A | 0.25 |
| **^α^** P+ indicates plasmids containing the native *ISAba125* promoter.  **^β^**AMP: Ampicillin, CEP: Cephalothin, FOX: Cefoxitin, CAZ: Ceftazidime, ERT: Ertapenem, IMP: Imipenem, DOR: Doripenem, MEM: Meropenem.  The shaded regions show data obtained from this study while the unshaded sections represent data obtained from previous studies^2,5,6,9,12,13,14,16^. | | | | | | | | | |

# Figure S6. Comparative MICs of *E. coli* TOP10 transformants.


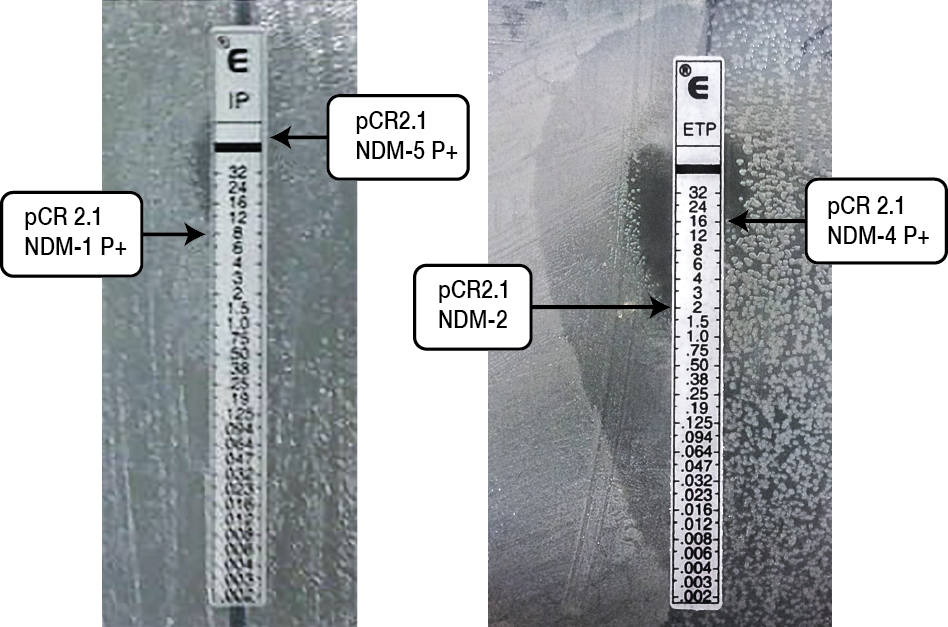
Comparison of susceptibility to Imipenem (IP) and ertapenem (ETP) of *E. coli* TOP10 illustrating the effects of the native promoter on the resistance profile. *E. coli* TOP10 cells were transformed with pCR2.1 NDM-1 P+, pCR2.1 NDM-5 P+, pCR 2.1 NDM-2 and pCR 2.1 NDM-4 P+ plasmids.

Table S7. Comparison of kinetic parameters from this study with literature values for NDM-1, NDM-3, NDM-4 and NDM-8.

| **Substrate** | **NDM-1**^16^ | | | **NDM-3**^5^ | | | **NDM-4**^6^ | | | **NDM-8**^14^ | | |
| --- | --- | --- | --- | --- | --- | --- | --- | --- | --- | --- | --- | --- |
|  | K_M_ (μM) | *k_cat_* (s^−1^) | *k_cat_*/K_M_ (s^−1^/μM) | K_M_  (μM) | *k_cat_*  (s^−1^) | *k_cat_*/K_M_ (s^−1^/μM) | K_M_  (μM) | *k_cat_* (s^−1^) | *k_cat_*/K_M_  (s^−1^/μM) | K_M_ (μM) | *k_cat_*  (s^−1^) | k_cat_/K_M_ (s^−1^/μM) |
| Imipenem | 94 | 20 | 0.20 | 148 ± 13 | 25 ±1 | 0.17 | 86 | 40 | 0.46 | 167 ± 8 | 46 ± 2 | 0.28 |
| This study | 78 ± 4 | 600 | 7.6 | 82 ± 12 | 757 | 9.2 | 62 ± 8 | 252 | 4.1 | 19 ± 3 | 54 | 2.9 |
| Meropenem | 49 | 12 | 0.25 | 81 ± 4 | 32 ± 1 | 0.4 | 95 | 30 | 0.31 | 127 ±20 | 169 ± 12 | 1.3 |
| This study | 57 ± 9 | 301 | 5.2 | 58 ± 11 | 238 | 4.2 | 119 ± 9 | 583 | 4.9 | 54  ± 5 | 142 | 2.6 |
| Ampicillin | 22 | 15 | 0.66 | 228 ± 35 | 73 ± 7 | 0.32 | ND | ND | ND | 193 ± 6 | 158 ± 5 | 0.82 |
| This study | 110 ± 23 | 447 | 4.1 | 258 ± 54 | 724 | 2.8 | 305 ± 48 | 900 | 2.9 | 229 ± 45 | 273 | 1.2 |
| Cephalothin | 10 | 4 | 0.4 | ND | ND | ND | 46 | 24 | 0.5 | ND | ND | ND |
| This study | 29 ± 6 | 75 | 2.6 | 13 ± 3 | 53 | 4.6 | 15 ± 3 | 25 | 1.6 | 5 ± 1 | 9 | 1.9 |
| Cefoxitin | 49 | 1 | 0.02 | 17 ± 1 | 1.6 ± 0.2 | 0.1 | NH | NH | NH | 34 ± 1 | 3 ± 0.1 | 0.1 |
| This study | 43  ± 6 | 10 | 0.23 | 50 ± 6 | 6 | 0.13 | 63 ± 7 | 14 | 0.22 | 16 ± 3 | 3 | 0.18 |
| Ceftazidime | 181 | 5 | 0.03 | 64 ± 9 | 11 ±9 | 0.17 | 72 | 4 | 0.06 | 63 ± 3 | 12 ± 0.2 | 0.2 |
| This study | 100 ± 19 | 24 | 0.23 | 115 ± 15 | 23 | 0.2 | 133 ± 18 | 28 | 0.21 | 19 ± 5 | 5 | 0.26 |
| NH: Not hydrolysed; ND:Not determined ; The shaded regions show data obtained from this study while the unshaded sections represent data obtained from previous studies .^5,6,14,16^ | | | | | | | | | | | | |

Figure S7. View from a structure of NDM-1 **complexed with hydrolysed methicillin (PDB code 4EYL)** ^17^**showing the proximity of Met-154 side chain with the aromatic ring of the substrate sidechain.**


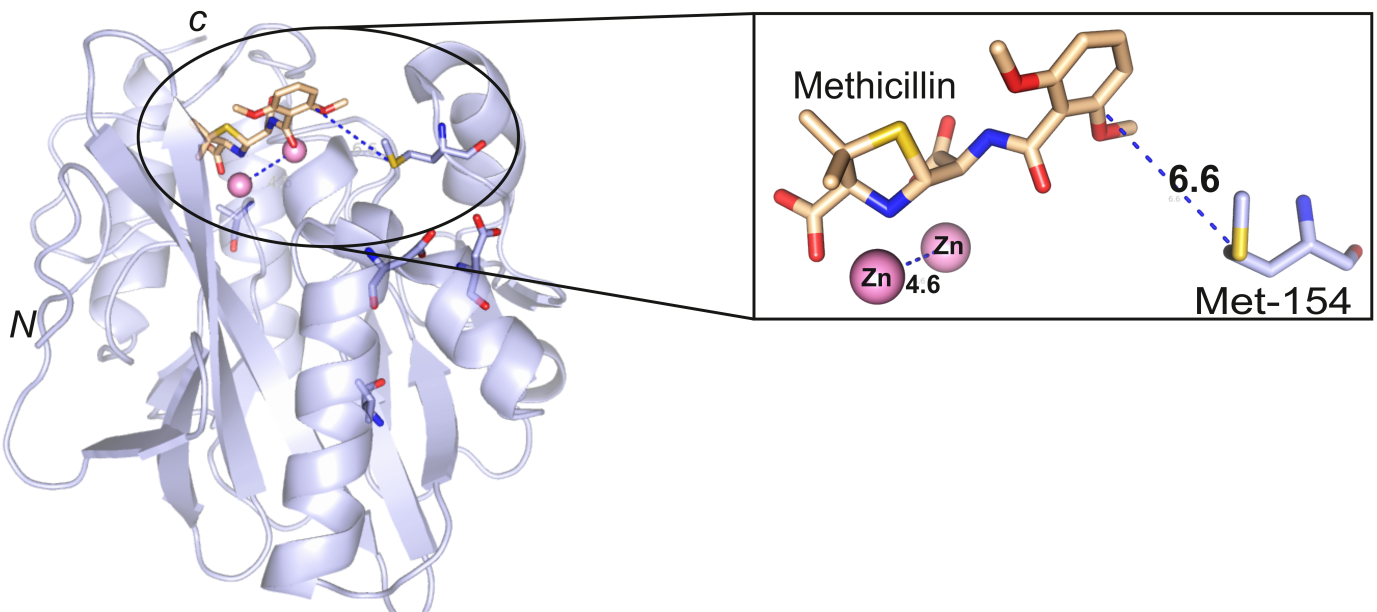


**References**

1. Gouet P, Robert X, Courcelle E. ESPript/ENDscript: Extracting and rendering sequence and 3D information from atomic structures of proteins. *Nucleic Acids Res* 2003; **31**: 3320-3.
2. Espinal P, Fugazza G, López Y *et al*. Dissemination of an NDM-2-producing *Acinetobacter* *baumannii* clone in an Israeli rehabilitation center. *Antimicrob Agents Chemother* 2011; **55**: 5396-8.
3. Espinal P, Poirel L, Carmeli Y *et al*. Spread of NDM-2-producing *Acinetobacter baumannii* in the Middle East. *J Antimicrob Chemother* 2013; **68**: 1928-30.
4. Rogers BA, Sidjabat HE, Silvey A *et al*. Treatment options for New Delhi Metallo-β-lactamase-harboring enterobacteriaceae. *Microb Drug Resist* 2013; **19**: 100-3.
5. Tada T, Miyoshi-Akiyama T, Shimada K *et al*. Biochemical analysis of the metallo-β-lactamase NDM-3 from a multidrug-resistant *Escherichia coli* strain isolated in Japan. *Antimicrob Agents Chemother* 2014; **58**: 3538-40
6. Nordmann P, Boulanger AE, Poirel L. NDM-4 Metallo-β-lactamase with increased carbapenemase activity from *Escherichia coli*. *Antimicrob Agents Chemother* 2012; **56**: 2184-6.
7. Jakobsen L, Hammerum AM, Hansen F *et al*. An ST405 NDM-4-producing *Escherichia coli* isolated from a Danish patient previously hospitalized in Vietnam. *J Antimicrob Chemother* 2014; **69**: 559-60.
8. Papagiannitsis CC, Studentova V, Chudackova E, *et al*. Identification of a New Delhi Metallo-β-lactamase-4 (NDM-4)-producing *Enterobacter cloacae* from a Czech patient previously hospitalized in Sri Lanka. *Folia Microbiol* 2013; **58**: 547–49.
9. Hornsey M, Phee L, Wareham DW. A novel variant, NDM-5, of the New Delhi Metallo-β-lactamase in a multidrug-resistant *Escherichia coli* ST648 isolate recovered from a patient in the United Kingdom. *Antimicrob Agents Chemother* 2011; **55**: 5952-4.
10. Rasheed JK, Kitchel B, Zhu W *et al.* New Delhi Metallo-β-lactamase-producing Enterobacteriaceae, United States. *Emerg Infect Dis* 2013; **19**: 870-8.
11. Williamson DA, Sidjabat HE, Freeman JT *et al*. Identification and molecular characterisation of New Delhi metallo-β-lactamase-1 (NDM-1)- and NDM-6-producing *Enterobacteriaceae* from New Zealand hospitals. *Int J Antimicrob Agents* 2012; **39**: 529-33.
12. Göttig S, Hamprecht AG, Christ S *et al*. Detection of NDM-7 in Germany, a new variant of the New Delhi metallo-β-lactamase with increased carbapenemase activity. *J Antimicrob Chemother* 2013; **68**: 1737-40.
13. Cuzon G, Bonnin RA, Nordmann P. First identification of novel NDM carbapenemase, NDM-7, in *Escherichia coli* in France. *PLoS One* 2013; **8**: e61322.
14. Tada T, Miyoshi-Akiyama T, Dahal RK *et al*. NDM-8 metallo-β-lactamase in a multidrug-resistant *Escherichia coli* strain isolated in Nepal. *Antimicrob Agents Chemother* 2013; **57**: 2394-96.
15. . Tada T, Shrestha B, Miyoshi-Akiyama T *et al*. A novel NDM variant, NDM-12, from a carbapenem-resistant *Escherichia coli* clinical isolate in Nepal. *Antimicrob Agents Chemother*. 2014. doi: AAC.03355-14.
16. Yong D, Toleman MA, Giske CG *et al*. Characterization of a new metallo-β-lactamase gene, *bla*_NDM-1_, and a novel erythromycin esterase gene carried on a unique genetic structure in *Klebsiella pneumoniae* sequence type 14 from India. *Antimicrob Agents Chemother* 2009; **53**: 5046-54.
17. King DT, Worrall LJ, Gruninger R *et al*. New Delhi metallo-β-lactamase: Structural insights into β-lactam recognition and inhibition. *J Am Chem Soc* 2012; **134**: 11362-5.
